# Elemental analysis and metabolic profiling of medicinally potent members of Zingiberaceae family using FT-IR and LIBS coupled with PLS-DA

**DOI:** 10.1016/j.heliyon.2024.e33395

**Published:** 2024-06-22

**Authors:** Saima Sohrab, Pratibha Mishra, Vishal Dwivedi, Pavel Veis, Ashok Kumar Pathak, Sanjay Kumar Mishra

**Affiliations:** a Department of Botany, Ewing Christian College, University of Allahabad, Prayagraj, Uttar Pradesh, 211003, India; b Photonics Laboratory, Physics Unit, Tampere University, Korkeakoulunkatu 3, 33720, Tampere, Finland; c Department of Experimental Physics, Comenius University, FMPI, Mlynská dol. F2, 842 48, Bratislava, Slovakia; d Department of Physics, Ewing Christian College, Prayagraj, 211003, Uttar Pradesh, India

**Keywords:** Zingiberaceae, Medicinal plants, *Curcuma*, *Zingiber*, *Alpinia*, *Costus*, FT-IR, LIBS, PLS-DA, Elemental analysis

## Abstract

The role of organic and inorganic elemental profiles in the growth, development, and secondary metabolite synthesis of plants is crucial, particularly concerning their medicinal value. However, comprehensive studies addressing both aspects are scarce. Hence, the present manuscript aims to investigate the potential use of Fourier transform infrared spectroscopy (FT-IR) and laser-induced breakdown spectroscopy (LIBS) techniques to obtain the functional groups and organic and inorganic elemental profiles of significant medicinal plants belonging to the Zingiberaceae family collected from two different geographic regions in India. The FT-IR analysis of the methanolic extracts shows the presence of aliphatic and aromatic alcohols, esters, ethers, carboxyl compounds, and their derivatives. In LIBS analysis, the spectral characteristics of atomic and molecular species present in the samples were observed, encompassing both organic and inorganic elements. The presence of heavy metals and trace elements have also been observed in the LIBS spectra of the samples. Furthermore, partial least squares discriminant analysis (PLS-DA) has been used to obtain classification pattern of the samples based on their spectral fingerprints. This study not only helps in reflecting the significance of micronutrients in aiding secondary metabolism thus enhancing the medicinal properties of plants, but also enables the identification of trace elements within plants. This facilitates the determination of the suitable usage and dosage of particular plant components, contributing to the research goal of establishing pharmacological and nutraceutical significance. This study is imperative as it fills a critical gap in research, although further work in this direction is warranted.

## Highlights

• FT-IR and LIBS were used for semi-quantitative studies on Zingiberaceae members.
• FT-IR has shown the presence of various ions, functional groups, and compounds.
• LIBS analysis has revealed organic and inorganic elements as well as trace elements.
• Samples were classified with a high accuracy of 93.14 % using PLS-DA with LIBS.

## Graphical abstract

**Figure:**
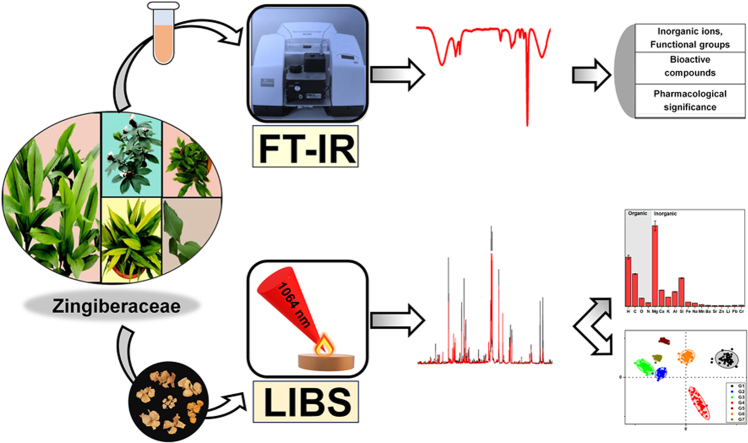

## 1 Introduction

Over the years, the ever-growing population of the world has witnessed various deadly endemic and pandemic outbreaks of microbial contagious diseases. Among these, viral infections like severe acute respiratory syndrome (SARS), dengue, human immunodeficiency virus (HIV), rotaviruses, respiratory syncytial, chikungunya, and influenza viruses have created havoc these days. Due to these infectious diseases, the innate and adaptative immunity of the body gets impaired [1]. It has been reported that certain macro- and micro-nutrients play significant roles in various immune responses. For example, maintaining the structural and functional integrity of the physical barriers of the immune system, chemotaxis, supporting the activity of anti-microbial proteins, and phagocytic activities of macrophages and neutrophils. These responses thus help in reducing the susceptibility of these infectious diseases and boosting the defense power of the body [2]. For instance, T-cell development, growth, and interferon gamma (INF-γ) production are all significantly influenced by iron [3]. Zinc is well recognized for its potential role in regulating host defense systems, particularly in viral illnesses, and its supplementation may even aid in the prevention of infection caused by SARS-CoV-2 [4].

Certain elements work as cofactors for enzymes and stimulate the activity of the protein. For example, selenium unifies with protein and forms selenoproteins, which act as antioxidants and help fight infectious pathogens by stimulating leukocytes, natural killer cells, interferons, and T-helper cells and also regulate the production of antibodies [5]. Besides, the immune system is also quite vulnerable to oxidative stresses, as the functional effectiveness of immune cells strongly depend on cellular contacts, particularly across membrane-bound receptors. Due to the peroxidation in the cell membrane entity, the integrity and fluidity of the membrane get impaired, and consequently, exposure and production of reactive oxygen species by the phagocytic cells get increased, causing the cells to damage themselves if not adequately protected by the antioxidants. Thus, antioxidants play a vital role in reducing and controlling the oxidative stress and infectious diseases [6]. Synthetic antioxidants and nutritional supplements used as immunity boosters nowadays possess harmful side effects due to the presence of artificial preservatives in them, and added to this, the inadequacy, extortionate prices, and inefficiency of synthetic drugs for curing various infectious diseases have aggravated the situation [7]. Thus, switching to herbal drugs is a cost-effective and safer alternative, as traditional medicines have served as a natural source of antimicrobial, antioxidant, and immunity boosters for ages. This can be evidenced by the fact that approximately 80% of the global population depends on medicinal plants to treat various illnesses, and their employment in herbal crude drug formulations [8,9]. The therapeutic values of the plants are affected by the phytoingredients, including bioactive components as well as mineral elements present in them [10]. Therefore, along with the phytochemical screening, the determination of the concentration and type of trace elements and heavy metals is equally crucial, not only because of their nutritional benefits but also to obtain an insight into the potential toxicity of the plants before applying them for therapeutic purposes. Taking this into account, some medicinally potent members of the family Zingiberaceae have been selected for this study.

The medicinal values of the family Zingiberaceae are widely recognized, and it has been described as one of the largest families in the plant kingdom, having about 50 genera and 1600 known species with their distribution in tropical regions of both hemispheres, throughout tropical Africa, Asia, and the Americas. India has 22 genera and about 170 species and is considered one of the richest and the most diverse regions for Zingiberaceae. The Northeast region of India harbors the greatest concentration, where 19 genera and about 88 species have been reported [[11], [12], [13]]. The ginger family constitutes a vital group of rhizomatous aromatic medicinal plants that have a very significant medicinal history in the ethnobotanical systems of traditional Chinese medicine, Ayurveda, and Western herbal medicine [14]. This family serves as an important natural source, providing varieties of products for food, spices, fragrances, dyes, medicines, aesthetics, and raw materials for uncountable domestic purposes for local communities for ages [15]. Furthermore, they are characterized by the presence of bioactive compounds of high medicinal value as they contain phenolic compounds, alkaloids, and majorly essential oils, including terpenes, alcohols, ketones, flavonoids, and phytoestrogens [16]. Zingiberene, curcumene, α- and β-turmerone, farnesene, α- and β-pinene, geraniol, borneol, citronellol, linalool, 1,8-cineole, camphor, methyl eugenol, β-bisabolene, β-sesquiphellandrene, camphene, myrcene, α- and β-phellandrene, limonene, α-terpineol, geranial, bornyl acetate, citronellyl acetate, α-copaene, α-terpineol and geranyl acetate etc., are some of the important phytocompounds exhibited by the family [[17], [18], [19], [20]]. Due to such bioactive compound's richness, the family possesses significant pharmacological activities, such as anti-bacterial, anti-inflammatory, anti-viral, anti-fibrotic, anti-cancer, hypocholesterolemic, anti-diabetic, anti-rheumatic, anti-hepatotoxic, gastroprotective, anti-arthritic, anti-oxidant, cardio protective, and neuroprotective activities [16]. In addition to the extensive list of pharmacological significance, the family also exhibits a rich mineral profile; for example, Zn, Cu, Ni, Ca, Mg, and Fe has been reported to be present in the rhizomes of *Zingiber officinale* and *Alpinia allughas* [21]. Similarly, the multi-elemental profiling of seven species, viz. *Zingiber zerumbet, Zingiber rubens, Curcuma zedeoaria, Kaempferia galanga, Kaempferia rotunda, Costus speciosus, Hedychium flavescens* revealed that these plants can be a reliable source of Mg, Mo, Cu, Na, and Zn [22].

However, there is a considerable information deficit regarding the elemental profile of potent medicinal plants, which should be researched imperatively as it reflects the physiological and pharmacological aspects of the plants. To address this, Fourier transform infrared spectroscopy (FT-IR) and laser-induced breakdown spectroscopy (LIBS) have been used in the current study. FT-IR spectroscopy is an analytical technique that involves the exposure of the samples to infrared radiation causing vibrations within the molecules of the samples due to the specific energy absorption and/or transmittance. This process aids in the evaluation of phytocomponents, particularly the characterization and identification of functional groups. A detailed description of the FT-IR method can be found in the literature [[23], [24], [25]]. Furthermore, for the rapid and reliable determination of multi-elemental composition, LIBS stands out as a robust and highly promising technique where the intensity of distinctive emission lines obtained can be directly linked to the elemental concentration in the composition. A comprehensive description of the LIBS method can be found in the existing literature [[26], [27], [28]]. Briefly, in LIBS, a high-energy laser pulse is directed and focused onto the surface of samples (regardless of the physical state of the samples), resulting in plasma formation after atomization and vaporization of a tiny amount of material. Characteristic emission from cooling plasma is then collected to obtain relevant spectral-based information about the constituents of the samples.

In the present work, elemental analysis and metabolic profiling of the medicinally potent plants of the family Zingiberaceae have been performed using FT-IR and LIBS spectroscopic methods. As a result of the information-rich characteristics of the LIBS method, spectral data was combined with chemometric technique, partial least squares-discriminant analysis (PLS-DA), to produce a classification pattern among samples. To the extent of our understanding, this is the very first FT-IR and LIBS combined analysis of *Alpinia galangal*, *Costus speciosus*, *Zingiber zerumbet*. Such studies on *Curcuma longa* and *Curcuma caesia* are present [29,30], but they differ in terms of plant collection sites.

## 2 Material and methods

### 2.1 Botanical description of the Zingiberaceae family and sample collection

The family Zingiberaceae contains a significant number of medicinal plants that are well-known for their use in ethnomedicine and play an important part in the Indian System of Medicine, Ayurveda [31]. Zingiberaceae members are generally aromatic, annual or perennial rhizomatous herbs with sympodially branched rhizomes in distinctive shapes and segments. The color of the rhizomes may vary from pale yellow to intense yellow, orange, blackish blue, greenish blue, pink, or other different characteristic color combinations in different species. The pseudo-stem has leaf sheaths, radial or cauline leaves, and a lamina with a strong central nerve, arranged distichously either parallel or transverse to the rhizomes. A short petiole may or may not be present. The flowers are hermaphrodite, zygomorphic, solitary, or cyme in the axils of the bract or on the peduncle with clasping sheaths, arising from the rhizome separately. The perianth is serrated and superior with a free calyx, imbricate, or connate in an entire toothed or spathaceous tube. Corolla tubes may be short or long, containing petaloid, and connate inner segments free or adnate to the petaloid staminodes. Petals are 3 in number which are united with short lobes. The number of perfect stamens can range from one to five, while imperfect stamens are in the form of conspicuous labellum and staminodes. Carpels (3) are syncarpous, with the inferior ovary having a mono- or tri-locular, axile placentation with many ovules. A slender style with short stylodes is present. The stigma may be entire or sub-entire. Fruit can be a loculicidal capsule or indehiscent and fleshy. Seeds are endospermic arillate with starchy endosperm and small embryos [32,33].

**Fig. 1:**
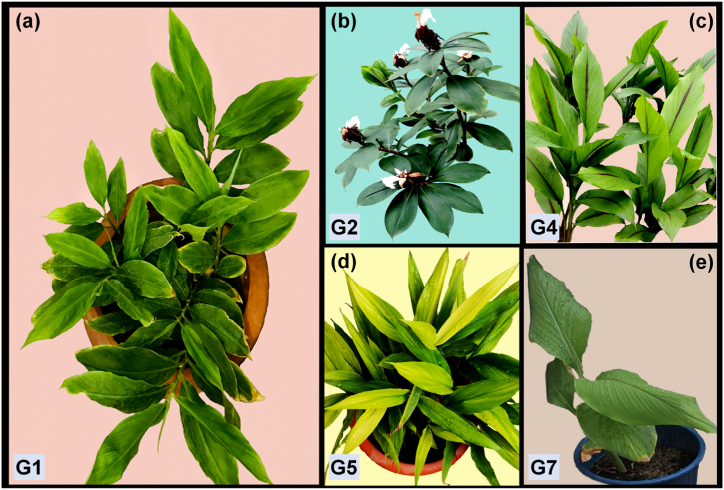
Photographs of medicinally important five members of the Zingiberaceae family (a) G1, (b) G2, (c) G4, (d) G5, and (e) G7.

Five medicinally important members of the family Zingiberaceae were collected, viz. *Curcuma caesia, Zingiber zerumbet* (from Assam, Northeast India), *and Curcuma longa, Costus speciosus, and Alpinia galangal* (from Uttar Pradesh, Allahabad, India), resulting in total seven samples. A summary of the sample nomenclature, local and botanical names, used parts, collection location, and the samples used for FT-IR and LIBS analysis, is provided in Table 1. In Fig. 1(a–e), photographs of five members of the Zingiberaceae family are shown.

**Table 1:** Details of the samples from Zingiberaceae family used in the study.

Sample ID	Local name	Botanical name	Used parts	Collection location	FT-IR	LIBS
**G1**	**Shampoo Ginger**	*Zingiber zerumbet*	Rhizome	Prayagraj	**✓**	**✓**
**G2**	**Spiral Ginger**	*Costus speciosus*	Rhizome	Prayagraj	**✓**	**✓**
**G3**	**Spiral Ginger**	*Costus speciosus*	Stem	Prayagraj	**✖**	**✓**
**G4**	**Black Turmeric**	*Curcuma caesia*	Rhizome	Assam	**✓**	**✓**
**G5**	**Galangal**	*Alpinia galangal*	Rhizome	Prayagraj	**✓**	**✓**
**G6**	**Galangal**	*Alpinia galangal*	Rhizome	Assam	**✖**	**✓**
**G7**	**Turmeric**	*Curcuma longa*	Rhizome	Prayagraj	**✓**	**✓**

For FT-IR analysis, 10 g of powder from the rhizomes of each of the selected members of the family Zingiberaceae (a total of five samples, G1, G2, G4, G5, and G7) were subjected to Soxhlet extraction using analytical-grade methanolic solvent at a temperature of around 78 °C and then filtered with Whatman filter paper having the pore size of 1 μm. Further, for the FT-IR study, translucent discs of the samples were prepared by encapsulating dried extract powder (10 mg) in a KBr pellet (100 mg). The spectra were recorded using KBr mode (spectral region: 4000-400 cm^−1^) using the PerkinElmer Spectrum Version 10.4.00 FT-IR spectrometer at Malaviya National Institute of Technology (MNIT), Jaipur, Rajasthan, India.

### 2.2 LIBS experimental scheme and measurements

**Fig. 2:**
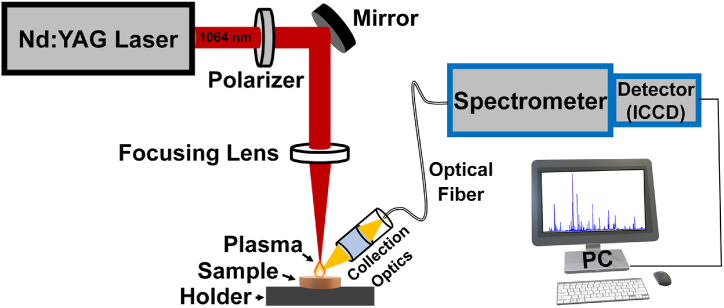
Schematic arrangement of the used LIBS set-up.

For LIBS analysis, the plant materials of all seven samples (G1 to G7), listed in Table 1, were thoroughly washed using double-distilled water and left for air drying at room temperature in a shady area. The samples were analyzed at the LIBS facilities of Comenius University, Bratislava, Slovakia, using a simple experimental scheme similar to that used in our previous works [34,35]. Briefly, for our experiment, a high energy Q-switched Nd:YAG laser (Brilliant Eazy, Quantel, wavelength 1064 nm) was used as a source, with a pulse duration of 5 ns, a repetition rate of 2 Hz, and an optimized energy of 10 mJ per pulse. The laser energy was controlled through the use of a polarizer and maintained throughout the measurements. The samples were placed on the X–Y manual translation stage, and the laser spots on the samples were varied to avoid overlapping of laser craters and to reduce heterogeneity effects. The laser beam was directed and focused on the sample surface with the aid of a mirror (Thorlabs, Nd:YAG mirror) and a plano-convex lens (Thorlabs, f = 100 mm). Laser-induced plasma optical emission was collected through collection optics and an optical fiber (0.5 NA and 1000 μm core diameter) and fed into an Echelle spectrograph (ME5000, Andor Technology, resolving power 4000, spectral range 200–850 nm) coupled with an intensified charge-coupled device (ICCD, iStar DH734, Andor Technology, temporal resolution 5 ns). This type of time-gated signal collection allows the user to select the suitable time window for signal collection and reject the unwanted strong continuum emission from the plasma. Fig. 2 shows the schematic representation of the used experimental set-up.

All the LIBS measurements have been performed under ambient air at atmospheric pressure and room temperature conditions. The optimized acquisition time delay (*t*_d_) and gate width (*t*_g_) were set to 500 ns and 2 μs, respectively, considering the optically thin plasma and local thermodynamic equilibrium (LTE) conditions. Each sample was subjected to a total of fifty spectra, with each spectrum being obtained by accumulating five laser shots at a single spot. This accumulation process aided in improving the signal-to-noise ratio. The samples were moved 500 μm after collecting spectrum from one spot to obtain a fresh spot for a new spectrum acquisition.

## 3 Results and discussion

### 3.1 FT-IR analysis

**Fig. 3:**
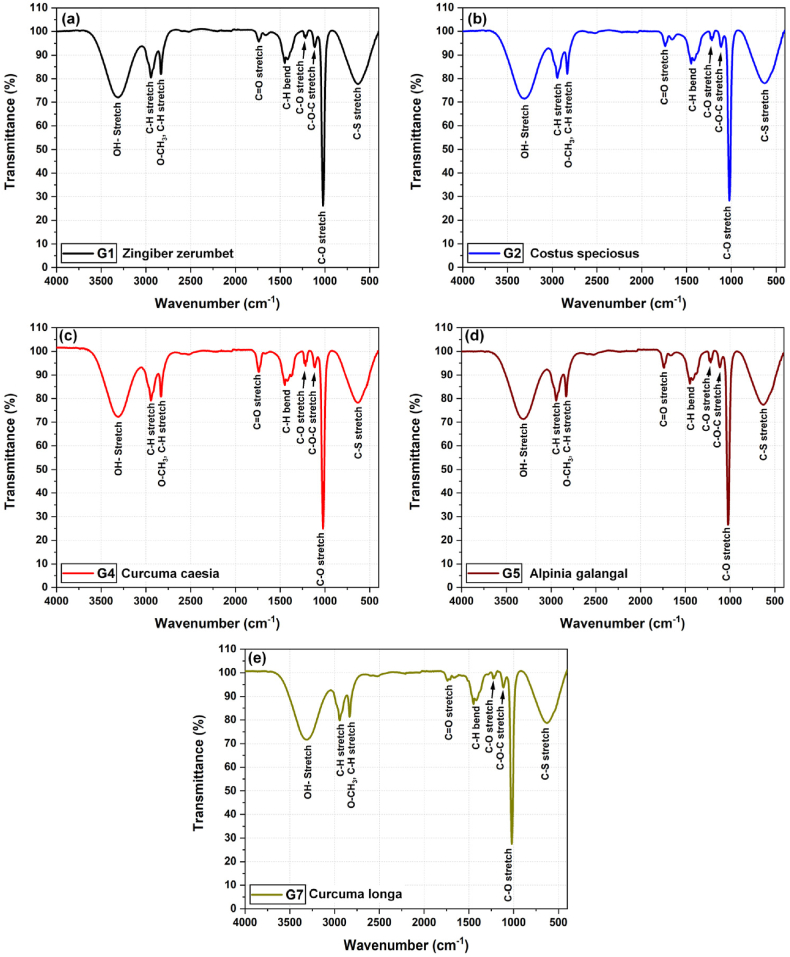
FT-IR spectra of methanolic extracts of members of the family Zingiberaceae (a) G1, (b) G2, (c) G4, (d) G5, and (e) G7, displaying distinct peaks for different functional groups signifying particular phytochemical compounds.

FT-IR analysis has been conducted to discern the various functional groups bound to the bioactive compounds present in the plant extracts. The graphical representation of the FT-IR spectra is presented in Fig. 3(a–e), and functional groups assigned to each peak have been reported in Table 2. The evaluation of the FT-IR results discloses three major peaks in the single bond region ranging from 4000 to 2500 cm^−1^. The broad absorption peak in the range 3570-3200 cm^−1^ revealed the presence of O–H stretch, and the peak at around 1021 cm^−1^ confirmed the C–O stretch. In the current investigation, aliphatic alkyl compounds were visible in the accompanying peak in the FT-IR spectrum range of 2970-2950 cm^−1^. The methyl group frequently exhibits such strong bands as a result of the symmetrical and asymmetrical stretching of the C–H modes [36]. The C–H stretch of ether (O–CH_3_) was represented by the peak at around 2831 cm^−1^ which was confirmed by the presence of a peak at around 1115 cm^−1^ reflecting the C–O–C stretch of ether. Another peak was obtained in the range of 1750-1725 cm^−1^ indicating the presence of carbonyl group (C=O), which suggests the presence of esters, while the C–O stretch was represented by the peaks obtained in the range 1150–1000 cm^−1^. A moderate peak at 635 cm^−1^ suggested the presence of thio or thioethers compounds (CH_2_–S-) as well as C–S stretching. The spectral signatures indicated that the selected members of Zingiberaceae most likely have conjugated dienes, including methyl, aliphatic chloro-compounds, nitrile, thiol, or thioether compounds. These results were found in agreement with the previous research done on Zingiberaceae members such as G2 [37], G4 [38,39], and G5 [40]. These results suggest the presence of alcohols, terpenes, esters, ethers, and thioether compounds. The compounds with an OH group have the ability to donate hydrogen atom or accept electron, scavenge free radicals, and also modulate the gene expression by interfering with the signaling cascade [41]. Terpenes are known to exhibit apoptotic, anti-metastatic, anti-inflammatory, and anti-angiogenesis activity because they show inhibitory action against TNF-α, NFK-β, and cytokines such as IL-6 [42]. Esters are also present in good amount in the Zingiberaceae members, exhibiting anti-flammatory activity, and helping in maintaining healthy lipid levels in the blood [43].

**Table 2:** FT-IR analysis of the selected members of the family Zingiberaceae ($\bar{v}$: Wavenumber, T: Transmittance).

Observed peak values										Functional groups
G1		G2		G4		G5		G7		
$\bar{v}$ (cm^−1^)	T (%)	$\bar{v}$ (cm^−1^)	T (%)	$\bar{v}$ (cm^−1^)	T (%)	$\bar{v}$ (cm^−1^)	T (%)	$\bar{v}$ (cm^−1^)	T (%)	
3307.3	72.01	3307.0	71.41	3307.6	72.26	3307.0	71.60	3307.0	71.60	O–H stretch
2943.9	80.48	2948.0	80.64	2948.6	79.20	2943.9	79.82	2943.9	79.82	C–H stretch
2831.8	81.92	2831.0	82.12	2831.6	80.72	2831.9	81.30	2831.9	81.30	O–CH_3_, C–H stretch
1738.3	95.78	1737.0	93.78	1737.6	91.24	1737.4	96.63	1737.4	96.63	C=O stretch
1448.7	86.58	1448.0	86.33	1448.5	85.74	1448.7	86.75	1448.7	86.75	C–H bend
1216.9	97.26	1216.0	96.32	1217.0	93.92	1229.6	97.58	1229.6	97.58	C–O stretch
1115.1	93.38	1115.0	93.36	1115.2	93.27	1115.2	93.72	1115.2	93.72	C–O–C stretch
1021.8	26.21	1022.0	28.30	1022.0	24.89	1022.0	24.89	1021.7	27.51	C–O stretch
631.7	77.73	634.0	78.02	634.9	78.32	626.5	78.65	626.5	78.65	C–S stretch

### 3.2 LIBS analysis

#### 3.2.1 Spectral analysis

**Fig. 4:**
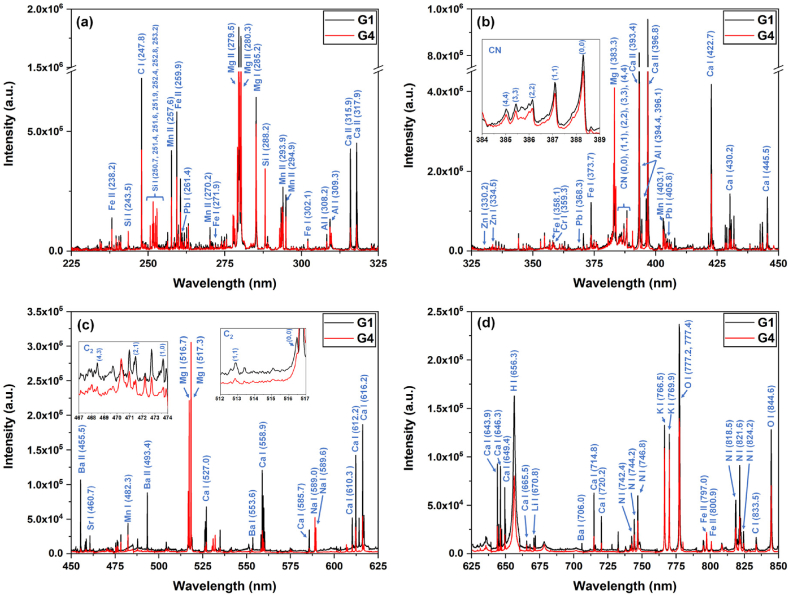
An example of the LIBS spectra for G1 and G4 samples for the wavelength range of (a) 225–325 nm, (b) 325–450 nm, (c) 450–625 nm, and (d) 625–850 nm. Inset images in figure (b) and (c) show the magnified section of the spectra related to CN and C_2_ molecular bands.

The LIBS analyses have been performed on all samples listed in Table 1. In Fig. 4(a–d), the LIBS spectra of G1 and G4 samples are presented for different wavelength ranges: 225–325 nm, 325–450 nm, 450–625 nm, and 625–850 nm. These spectra are the average of 50 individual spectra (each individual spectrum was the accumulation of 5 laser shots) from the same sample and have been corrected with the spectral response of the used spectrograph. The LIBS spectra, as shown in Fig. 4(a–d), exhibit atomic neutral and ionic spectral lines corresponding to both organic (H, C, O, and N) and inorganic elements (Mg, Ca, K, Al, Si, Fe, Na, Mn, Ba, Sr, Zn, Li, Pb, and Cr). The identification of the spectral lines has been done using NIST atomic spectral database [44] and the spectral lines used to identify elements are summarized in Table 3. The most abundant elements are the organic elements which are essential components of the organic compounds found in these medicinal plants, such as carbohydrates, proteins, and lipids. It is worth noting that the measurements were conducted in air atmosphere, and it is possible that atmospheric constituents may have influenced the spectroscopic signals of organic elements. Along with organic elements, the presence of minerals in low or trace amounts contributes to the overall nutritional profile of the plants. In addition to the atomic spectral lines, the presence of CN Violet (B^2^Σ^+^ to X^2^Σ^+^) and C_2_ Swan (d^3^П_g_ - a^3^П_u_) molecular bands further confirms the presence of carbon-containing compounds and the organic nature of the samples [45]. The magnified sections of the spectra corresponding to the CN and C_2_ molecular bands are presented as inset images in Fig. 4(b) and (c).

**Table 3:** List of the elements and corresponding spectral lines present in LIBS spectra. Bold marked lines were further used for estimating elemental intensity variations in section 3.2.2. (*I: neutral atomic, II: ionic).

S. No.	Elements		I/II*	Wavelength of spectral lines (nm)
**1**	**Organic**	Hydrogen (H)	I	**656.3**
**2**		Carbon (C)	I	**247.8**, 833.5
**3**		Oxygen (O)	I	777.2, 777.4, **844.6**
**4**		Nitrogen (N)	I	742.4, 744.2, **746.8**, 818.5, 821.6, 824.2
**5**	**Inorganic**	Magnesium (Mg)	I	**285.2**, 383.3, 516.7, 517.3
			II	279.5, 280.3
**6**		Calcium (Ca)	I	**422.7**, 430.2, 445.5, 527.0, 558.9, 585.7, 610.3, 612.2, 616.2, 643.9, 646.3, 649.4, 665.5, 714.8, 720.2
			II	315.9, 317.9, 393.4, 396.8
**7**		Potassium (K)	I	766.5, **769.9**
**8**		Aluminum (Al)	I	308.2, **309.3**, 394.4, 396.1
**9**		Silicon (Si)	I	243.5, 250.7, 251.4, 251.6, 251.9, 252.4, 252.8, 253.2, **288.2**
**10**		Iron (Fe)	I	271.9, **302.1**, 358.1, 373.7
			II	238.2, 259.9, 797.0, 800.9
**11**		Sodium (Na)	I	**589.0**, 589.6
**12**		Manganese (Mn)	I	403.1, **482.3**
			II	257.6, 270.2, 293.9, 294.9
**13**		Barium (Ba)	I	**553.6**, 706.0
			II	455.5, 493.4
**14**		Strontium (Sr)	I	**460.7**
**15**		Zinc (Zn)	I	330.2, **334.5**
**16**		Lithium (Li)	I	**670.8**
**17**		Lead (Pb)	I	261.4, **368.3**, 405.8
**18**		Chromium (Cr)	I	**359.3**
**19**	**Molecular bands**	CN Violet		**(0, 0): 388.3, (1, 1): 387.1, (2, 2): 386.1,** **(3, 3): 385.4, (4, 4): 385.0**
**20**		C_2_ Swan		**(0,0): 516.5, (1, 1): 512.9,** **(1, 0): 473.7, (2, 1): 471.6, (4, 3): 468.3**

#### 3.2.2 Elemental intensity variation

Based on spectral analysis, multiple organic and inorganic elements were identified in the samples, as listed in Table 3. These elements contribute significantly to the nutritional and medicinal values of the plants. In LIBS, the observed intensity of spectral lines emitted by elements is found to be directly correlated with the concentration of those elements present in the samples. However, for the accurate determination of an absolute elemental concentration, it is required to establish a calibration curve using a standard reference material with a known concentration [46]. This requirement poses practical challenges when analyzing plant samples due to the unavailability of suitable calibration standards. In order to gain semi-quantitative insight into the elemental concentrations in the samples, we have extracted the integral intensity of one prominent and strong (in terms of signal-to-noise ratio) spectral line for each element (highlighted in bold in Table 3). While some of the selected spectral lines are resonant lines, we have ensured that they are free from self-absorption by comparing them with other non-resonant spectral lines. Spectral regions specific to each line of interest were isolated from full-range spectra to obtain their integral intensities. These regions underwent background subtraction, and the spectral lines were fitted with a pseudo-Voigt function to determine their integral area, which we refer to as their integral intensity [47,48]. These intensities were further normalized relative to the maximum intensity among all the elements, scaling the maximum value of integral intensities to 1 for better visualization. Furthermore, the verification of the LTE condition was conducted using the McWhirter criterion [49,50]. Plasma temperature was determined from Boltzmann plots of Ca I spectral lines, while electron density was evaluated through the Stark broadening of the *H*_α_ spectral line at 656.3 nm. Among the samples, the observed plasma temperature ranged from 8,629 K to 12,106 K, while the electron density ranged from 5.6⋅10^17^ cm^−3^ to 6.8⋅10^17^ cm^−3^, validating the LTE conditions.

**Fig. 5:**
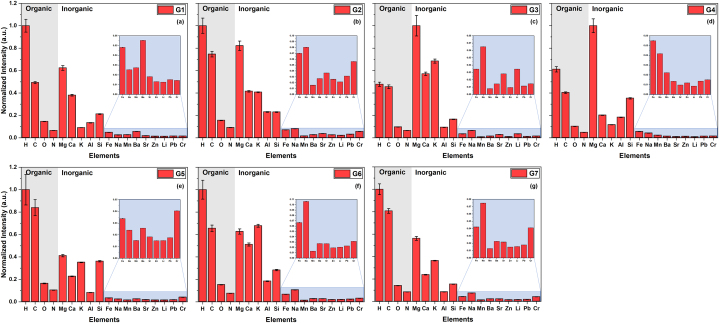
Element-wise normalized spectral line integral intensity variation for sample (a) G1, (b) G2, (c) G3, (d) G4, (e) G5, (f) G6, and (g) G7. In all the figures, an inset image has been included for better visualization of the elements with low intensity values. Organic and inorganic elements have also been marked. The error bars indicate the standard deviation of the data.

Fig. 5(a–g) shows the variation in spectral line intensities of all potential elements for all the analyzed samples. Fig. 5(a–g) reveals that among the organic elements, H exhibits the highest intensity, indicating its predominant presence. Additionally, C, O, and N also exhibit significant intensities, showcasing their considerable abundance in the present organic compounds. The overall trend of the variation in organic element intensities appears to be consistent across all samples. In case of inorganic elements, the significant presence of Mg, Ca, K, Al, and Si elements can be easily seen, while other elements, i.e., Fe, Na, Mn, Ba, Sr, Zn, Li, Pb, and Cr, are present in a very low or trace amount. It is worth noting that the Mg signal is stronger than the signal from H and C, in samples G3 and G4. The strong presence of Mg may be attributed to its accumulation in plant tissues (rhizomes and stems) from the soil, applied fertilizers, or other enzymatic reactions. Furthermore, the presence of other minerals and trace elements (especially toxic elements Li, Pb, and Cr) can be attributed to agricultural inputs such as water and fertilizers, soil characteristics, and environmental exposure. The presence of trace elements or heavy metals can have detrimental effects on the nutritional value of the plants, consequently posing risks to human health, therefore it is crucial to have control over such elements. In this regard, the utilization of the LIBS method in our work is highly beneficial for such elemental quality control purposes.

**Fig. 6:**
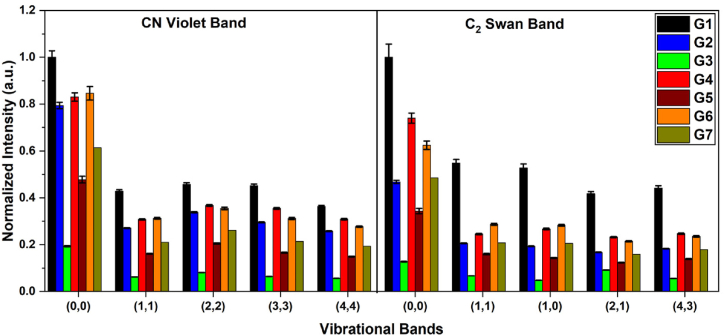
Normalized spectral line intensity variation of different vibrational transitions of CN Violet and C_2_ Swan bands for all the samples. The error bars indicate the standard deviation of the data. (For interpretation of the references to color in this figure legend, the reader is referred to the Web version of this article.)

The presence of CN and C_2_ molecular bands, as shown in Fig. 4(b and c), is generally arising from the combination of atoms during the ablation of samples and formation of molecules or from the dissociation of carbon-containing molecules. Analysis of these bands contributes to a comprehensive elemental analysis of the samples. Fig. 6 shows the variation of normalized intensities corresponding to different vibrational transitions for both CN Violet and C_2_ Swan bands, for all samples. In both cases, the lowest energy state (0, 0) is the most intense, with the presence of additional bands with lower intensities in all the samples. The intensities of these bands are significantly lower for samples G3 and G5 in comparison to other samples.

#### 3.2.3 Elemental correlation

**Fig. 7:**
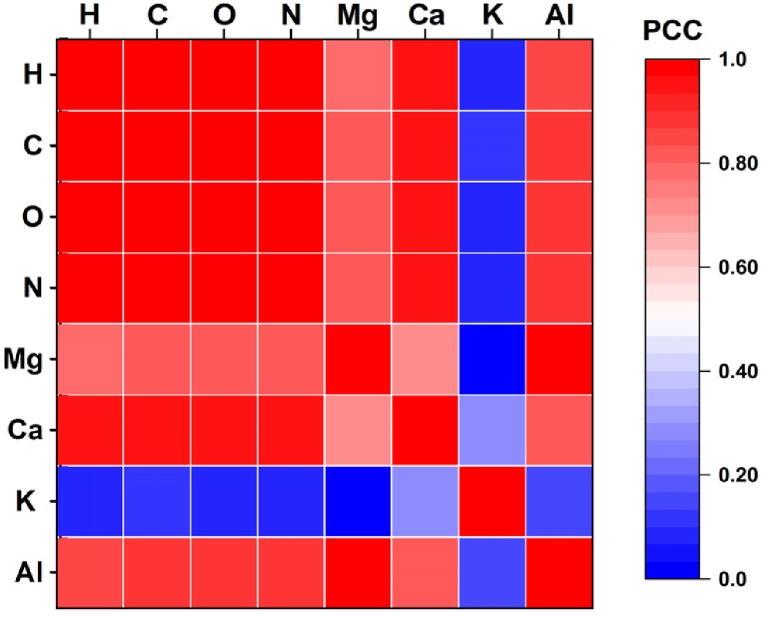
Spectral line intensity correlation plot of main elements present in the samples.

The correlation among the intensities of the main constituent elements has been established using the spectral line integral intensities. A similar approach has been performed [51] for a set of different sea salt samples. Fig. 7 shows the correlation plot of the intensities of different elements (H, C, O, N, Mg, Ca, K, and Al). The correlation has been established based on the Pearson correlation coefficient (PCC), which has a value between −1 and 1 (−1 indicates a strong negative correlation, 1 indicates a strong positive correlation, and 0 indicates that there is no correlation). As already stated in section 3.2.2, the organic elements have shown a consistent pattern of intensity variation for all the samples, indicating that they are highly correlated. This observation was further supported by Fig. 7, which demonstrated PCC values greater than 0.99 for the organic elements, indicating a strong positive correlation. In contrast, the correlation among inorganic elements was comparatively lower, with PCC values ranging from 0.72 to 0.96 for different element combinations. Notably, K displayed a lack of correlation with other elements, with PCC values ranging from 0 to 0.20. This behavior of K may be attributed to its origin, as in plants it is primarily derived from soil and other cultivation-related inputs.

#### 3.2.4 PLS-DA classification

Leveraging the information-rich nature of the LIBS method, the LIBS spectra acquired from all samples were subjected to PLS-DA for obtaining a classification pattern of the samples. PLS-DA is a supervised multivariate statistical method which is widely used for multi-class classification [52]. The fundamental principles of PLS-DA are extensively documented in literature [[53], [54], [55]]. PLS-DA accomplishes dimensionality reduction, feature selection, and discrimination while fully considering class labels. In brief, the method involves transforming the data matrix into a new matrix of reduced dimensions using a transformation matrix and an error matrix. The features representing the reduced dimensions in PLS-DA are referred to as principal components (PCs). PLS-DA relies on covariance calculation and iterative processes, utilizing the error term to define various PCs.

**Fig. 8:**
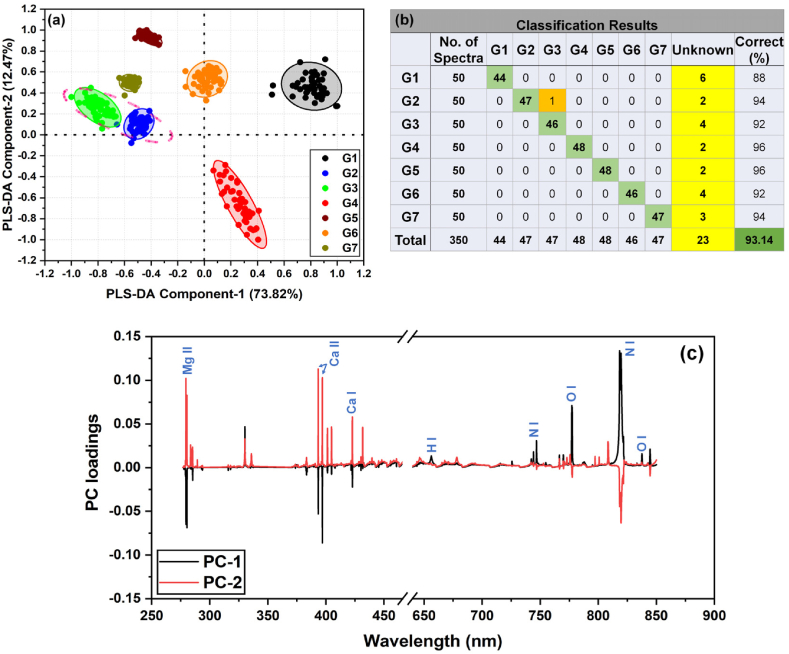
Normalized PLS-DA (a) score plot from first two components, (b) classification table, and (c) loadings plot for first two components, for all samples.

A total of 210 spectra (30 spectra per sample, each spectrum containing 23,400 variables or wavelengths), which were randomly selected, have been used for training the model. MATLAB-based scripts have been used to perform the analysis. The remaining 140 spectra (20 spectra per sample) were used to validate the model, and 134 of them were correctly classified, giving the model an accuracy of 95.7%. The six spectra that were not correctly classified are not misclassified to another sample class but rather are marked as unknown by the model. Furthermore, to test the robustness of the model, a classification pattern was generated by re-shuffling all 350 spectra. Fig. 8(a) shows a normalized score plot of all 350 spectra obtained from PLS-DA analysis. In the score plot, each data point represents an individual spectrum. Here, the min-max normalization technique has been employed to rescale the score plots within the range of −1 to +1. The 95% confidence ellipses have been derived by the Hotelling T^2^ method in order to detect misclassifications. The parameters for these ellipses are determined using covariance matrices and eigenvalues [56]. The cumulative percentage of first two PCs is 86.29% (component 1: 73.82%, component 2: 12.47%). In the score plot, all the samples were grouped separately and no overlap between the spectra of different samples was observed. Also, the groups from sample G2 and G3 are close to each other, because of their common origin within the Rhizome and Stem sections of the same plant. Notably, the identified outlier spectra differ from the rest of the sample spectra in some aspect. The possible factors contributing to these outliers include sample heterogeneity, sample surface contamination, and instrumental fluctuations.

Fig. 8(b) presents a table summarizing the classification results. It shows the number of spectra that were correctly classified into their respective sample classes (main diagonal values), as well as those that were either classified as unknown or misclassified (off-diagonal values) by the PLS-DA model. Out of 350 spectra, one was misclassified (i.e., a spectrum belonging to G2 class was identified as G3 class), and 23 spectra were marked as unknown. As a result, 93.14% of the classifications were correct. The observed variability within certain samples (such as G1 and G4) may be attributed to the heterogeneity of the sample matrices. Fig. 8(c) presents the loadings plot for the first two PCs for the spectral regions 275 nm–465 nm and 640 nm–850 nm. This plot illustrates which variables (wavelengths) contribute the most to the discrimination plot compared to other pixels.

Based on the results obtained in section 3.2, it is demonstrated that LIBS combined with chemometric method (PLS-DA) can be effectively used for the correct elemental identification and classification of samples based on their spectral differences.

## 4 Conclusions

The semi-quantitative elemental analysis and metabolic profiling of medicinally potent members of the family Zingiberaceae have been obtained using spectroscopic methods, FT-IR and LIBS coupled with PLS-DA. The current study on Zingiberaceae members shows the presence of phenolics, flavonoids, terpenes, etc., thereby reflecting the pharmacological potency of the plants. It can be concluded that this study also helps establish the phytochemical profile of the under-explored and endangered plant, *Curcuma caesia*. Noteworthy similarities have been found between its FT-IR spectra and those of other well-established medicinal plants like *Costus* and *Curcuma longa*, suggesting that *Curcuma caesia* can also be harnessed for its equivalent degree of therapeutic potential. Furthermore, it was found that even though the samples belong to the same family, they differ significantly in their elemental compositions. The presence of organic elements was further confirmed by LIBS analysis of the same samples. In addition to organic elements, inorganic elements, and minerals (i.e., Mg, Ca, K, Al, and others) have also been found in different proportions in different samples. These essential macro- and micro-nutrients can be correlated with the plant's pharmacological and nutraceutical potential. Zn acts as a cofactor for antioxidant enzymes such as superoxide dismutase, and it protects the plants from cellular abiotic stresses. Similarly, Mn accelerates the activity of enzymes engaged in the biosynthesis of pharmaceutically acclaimed secondary metabolites such as flavonoids, anthocyanins, and lignin, as enhancements in these bioactive compounds ultimately enrich the medicinal properties of the plants. This study also reveals that *Curcuma caesia* exhibits a higher magnesium concentration, suggesting its potential as a nutraceutical for boosting immunity, as magnesium aids in anti-inflammatory responses and boosts immunity. Besides, the utilization of the LIBS method in our work was found to be highly beneficial for detecting the presence of trace elements or heavy metals (Li, Cr, and Pb) that can have detrimental effects on the nutritional value of the plants, consequently posing risks to human health. It also explains that since these plants show bioaccumulation of trace or heavy metals, a thorough investigation regarding their elemental profile is necessary before using them in pharmaceutical applications. The classification of the samples using LIBS spectroscopic data as an input to the PLS-DA chemometric method was achieved with a correctness of 93.14%. Such studies on medicinal plants should be encouraged and must be addressed before their traditional as well as pharmaceutical applications, as they reflect a collective or multi-dimensional approach that gives important information regarding the phytochemical and nutritional profile as well as quality control aspects, including trace and heavy metals presence in the potent medicinal plants.

## Originality

The research presented in this manuscript is our original work. It has not been published previously, and it is not under consideration for publication elsewhere, in full or in part.

## Funding

V.D. and P.V. acknowledge the financial support from Scientific Grant Agency of the Slovak Republic (VEGA-1/0803/21) and the Slovak Research and Development Agency (APVV-22-0548).

## Data availability

**Table:** 

Has data associated with your study been deposited into a publicly available repository?	No
Has data associated with your study been deposited into a publicly available repository?	**Data will be made available on request**

## CRediT authorship contribution statement

**Saima Sohrab:** Writing – original draft, Validation, Methodology, Investigation, Formal analysis, Conceptualization. **Pratibha Mishra:** Investigation, Formal analysis, Data curation. **Vishal Dwivedi:** Writing – review & editing, Visualization, Software, Methodology, Investigation, Formal analysis, Data curation. **Pavel Veis:** Resources, Writing – review & editing. **Ashok Kumar Pathak:** Writing – review & editing. **Sanjay Kumar Mishra:** Writing – review & editing, Validation, Supervision, Resources, Project administration, Data curation.

## Declaration of competing interest

The authors declare that they have no known competing financial interests or personal relationships that could have appeared to influence the work reported in this paper.
